# Evaluation of the synthetic estimators with non-respondent for small domain

**DOI:** 10.1016/j.heliyon.2024.e29592

**Published:** 2024-04-15

**Authors:** Abdullah Mohammed Alomair, Ashutosh Ashutosh

**Affiliations:** a Department of Quantitative Methods, School of Business, King Faisal University, Al-Ahsa, 31982, Saudi Arabia; b Department of Statistics, Allahabad Degree College, University of Allahabad, Prayagraj, 211003, India

**Keywords:** Domain, Calibration approach, Supportive variable, Synthetic ratio estimator, 62D05

## Abstract

The present work gives generalized form of the class of estimators if unit non-response is acquired in small domains. We have obtained indirect method like synthetic. It has provided specific instances where the proposed estimators are unbiased. It has also given a generalized form of the synthetic estimators. The four estimators were investigated with coefficient of variation and kurtosis constants. Because, these are obtained from the available variable. We conducted atheoretical comparison between traditional and proposed calibration estimators for estimating the domain totals. It found that the proposed estimators are more efficient than traditional estimators for investigate small domains.

## 1 Introduction

The impact of supportive variable can show in the calibrated estimator. Calibration idea is one of the most prominent types of idea which utilized restriction of shortest distance. In small domain estimation, the use of supportive variable is contrasting ways to enhance superiority of estimators. The domains contain issue of the deviation in the present unit? How these units can be provide acceptable information. It may provide by the work in Refs. [[1], [2], [3]]. A pioneer contribution to estimate the population total with calibration idea was developed [4]. In this kinship, the impending for variance of ratio and generalized regression have been discussed in Refs. [5,6]. However, several contributions for estimation of variance using the calibration idea have discussed in references [7,8]. In couple of the years, the interest ofthe local area estimate is more preferred over the population information estimate due to the effectiveness regarding to policy implementation. Sometime these local areas are called small domain. In these small domains, accessible study on the small domain estimation plays an imminent role during frame of the programs and policies for both public and private utilities. Hence, in the present times, it is widely booming. Various studies about the small domain with its utility of supportive variable have been contributed in references [[9], [10], [11]]. Synthetic type estimator simply utilizes the surrounding value of the small domain (decide on size) for interested domain (study domain). Generally, synthetic estimator is used when small units access in the interested domain. While, it should not for apply if same unit present in all the domains. Two prominent reasons are: the absence of sufficient units in the domain under study characteristic and poor performance of direct estimation method. The favorable units do not obtain reliable results away for study domain. In small unit cases calibration estimation give permissible estimate. The supportive information utility gives another benefit that finished polish result for the synthetic idea. The synthetic estimator is a model based idea under the assumption that the small domain information is very near to the population estimation [12]. However, when some units may be lost due to instantaneous reason in small domain. The non-response problem can occur. The unit level value of the study variable can deal in the estimation when the no respondents are available in the study variable [13,14]. The non-respondent in the real situation may provide poor information. This problem can reduce efficiency of the estimator. In this type of the situation two twists (low units and non respondent in the domain) can handle, and it is convincing task during selection of domain. The non correspondent found in many ways which was estimated in Refs. [3,15]. Real problem faces by information of sum of auxiliary unit of the population is equal to the domain total. Because loss of information in ath domain and population weight of study character closely reach to auxiliary character restriction. reference [16] provides indirect method in the presence of the unit non response. This article provides weight adjustment with design and model based estimators. We recommend the specific works in Refs. [17,18] to get insights into the investigated estimators with advancements in presence of the unit non-respondent. A real example of non respondent is that if so many patients previous history of disease do not provide health practitioner on the time of accident.

The formulation of the problem mentioned in the methodology section of this paper. The main objective of the work is to deal the surrounding information which may benefit to the study domain.

The brief console of the present work is given as: Section 2, presents methodology of the calibration and subsection discusses review of direct and indirect ideas. Section 3 describes propose estimator of the domain total with a supportive variable in two cases. Section 4 describes conclusion of synthetic type estimators. Section 5 gives application of the work. Section 6 provides future plan of relevant work.

## 2 Methodology

Consider the non-overlapping $a^{th}$ small domains *U*_*a*_ with consisting of *N*_*a*_ unit. The value of sum of all the domains is $\sum_{a=1}^{M}N_{a}=N$, such that (a=1,2,............,.M). To estimates the parameter like total for $a^{th}$ domain. A unique population (U=1,2,............,....N) consists of all small domain that is represent by $U_{a}=1,2,............,....N_{a}$; $U_{a}\subset U$. A random sample *s* with *n* unit is selected by the population *S* of *N* unit by interesting variable y and supportive variable x (from U) and design weight is $d_{i}=\frac{1}{\pi_{i}}$. It is assumed unit n consisted of the respondent unit $n_{1}$ and non-respondent unit $n_{2}$. Whenever, we choose a random sample with $n_{2r}$ unit which is the portion of the non respondent unit $n_{2}$ that is $n_{2r}=n_{2}/l,l>1$. reference [19] developed a concept of estimation of non respondent technique for estimating the non-respondent on the based on unit $n^{*}=n_{1}+n_{2r}$. It is due to subsampling only by non respondent. Hence, it saves time, cost and efficiency of the estimator. The weight adjustments of an interest variable when non respondent are ongoing and given by Eq. (1)

(1) $$\sum_{i=1}^{n^{*}}t_{i}y_{i}^{*}=\sum_{i=1}^{n_{1}}t_{1i}y_{1i}+\sum_{i=1}^{{n_{2r}}_{1}}t_{1i}t_{2i}y_{2ri}^{\prime }\text{,}$$

and value of each unit of the supportive variable which correspond to interest variable is given by Eq. (2)

(2) $$\sum_{i=1}^{n^{*}}t_{i}x_{i}^{*}=\sum_{i=1}^{n_{1}}t_{1i}x_{1i}+\sum_{i=1}^{{n_{2r}}_{1}}t_{1i}t_{2i}x_{2ri}^{\prime }\text{.}$$

Study and supportive variables and their relevant notations are:

$N_{1}$: Total count of the response units in the population.

$N_{2}$: Total count of the non-respondent on the population.

Y: Total count of an interested variable y on *N* observations.

$Y_{a}$: Represent ath domain total

$y^{*}$: Sample value of an interested variable on n* observations.

$x^{*}$: Sample of a supportive variable based on n* observations.

$N_{1}$: Count of response units in the sample.

$N_{2}$: Count of non-response units in the sample.

$y_{1i}$: Respondent sample of y based on $n_{1}$ respondent units.

$x_{1i}$: Respondent sample of y based on $n_{1}$ respondent units.

$y_{2i}^{\prime }$: Non-respondent sample of y based on $n_{2r}$ respondent units.

$x_{2i}^{\prime }$: Non-respondent sample of y based on $n_{2r}$ respondent units.

### 2.1 Reviews of synthetic calibration

The calibration idea for estimation of the population total is based on reference [20] which is an unbiased estimator ${\hat{Y}}_{HT}=\sum_{i\in S}d_{i}y_{i}$. The value of the supportive variable with design weights and population total is $\sum_{i\in S}d_{i}x_{i}=X$. There are many distances. But in most of the cases chi-square provides better results in Ref. [16]. Hence, it has not gone to other distance. The value of X is the known population total of the x then the chi-square distance type function is $\varphi =\sum_{i\in S}\frac{{\left(d_{i}-p_{i}\right)}^{2}}{d_{i}q_{i}}$. Where, $q_{i}$ is a chosen constant either positive or negative? The probability $p_{i}$ is the new weights and using the Lagrange's multiplier. It gets a calibration estimator ${\hat{Y}}_{c}=\sum_{i\in S}p_{i}y_{i}={\hat{Y}}_{HT}+{\left(X-{\hat{X}}_{HT}\right)}^{\prime }\hat{B}$.

where, $\hat{B}={\left[\sum_{i\in S}d_{i}q_{i}x_{i}x_{i}^{\prime }\right]}^{-1}\sum_{i\in S}d_{i}q_{i}x_{i}y_{i}$, ${\hat{X}}_{HT}=\sum_{i\in S}d_{i}x_{i}$ and ${\hat{Y}}_{HT}=\sum_{i\in S}d_{i}y_{i}$ are Horvitz-Thompson estimators. However, in small area estimation, synthetic type generalized and ratio type estimators of total of ath domain is written by

(1) ${\hat{Y}}_{TG,a}={\hat{Y}}_{HT}+\hat{\beta }\left(X_{a}-{\hat{X}}_{HT}\right)$, where, $\hat{\beta }=\frac{\sum_{i=1}^{n}x_{i}y_{i}}{\sum_{i=1}^{n}x_{i}^{2}}$ and $\pi_{i}=\frac{n}{N}$.
(2) Synthetic type of ratio estimator for domain total is ${\hat{Y}}_{TR,a}={\hat{Y}}_{HT}\left(\frac{X_{a}}{{\hat{X}}_{HT}}\right)$.

Where, ${\hat{Y}}_{HT}=\sum_{i=1}^{n}\frac{y_{i}}{\pi_{i}}$, ${\hat{X}}_{HT}=\sum_{i=1}^{n}\frac{x_{i}}{\pi_{i}}$, $X_{a}=\sum_{i=1}^{N_{a}}X_{a\;i}$, and also $\pi_{i}=\frac{n}{N}$.

The two mentioned case (1) and case (2) and other types of the estimators with non responses were recently investigated in Ref. [16]. That work highlighted the utility of the nearer domain information which help us to improve the developed estimators for an intended domain.

## 3 Propose estimator

We initially start for utilization of the auxiliary variable which is a challenging task for estimation of the synthetic type ratio estimator with the help of the calibration idea [21,22]. Calibration is the utility of the helping variable and size of the unit available in the regions. We used the unit value which is value of variable which can not further subdivide. For example, in the domain there are i = 1, 2, 3, …, N units, so the 5th unit level value means value of the 5th position value in the population. The propose estimator of domain total with unit non-respondent is written in Eq. (3)

(3) $$T_{PS,a}=\sum_{i=1}^{n^{*}}t_{i}y_{i}^{*}\text{.}$$

A supportive variable is soleon the n* observations with the new weights. Domain total of the study domain of respondent and non-respondentis written by Eq. (4)

(4) $$\sum_{i=1}^{n^{*}}t_{i}x_{i}^{*}=X_{1a}+X_{2a}=X_{a}\text{.}$$

Where, $X_{a}$ is the known population total of x. The shortest distance is given as Eq. (5)

(5) $$D=\sum_{i=1}^{n^{*}}\frac{{\left(t_{i}^{*}-t_{i}\right)}^{2}}{t_{i}q_{i}}\text{,}$$

where, $q_{i}$ is chosen constants. The $t_{i}^{*}$ shows the new weight very near to the earlier weight $t_{i}$. The Lagrange multiplier equation under minimizing condition from Eq. (4) and to the calibration constraint Eq. (5) is as Eq. (6)

(6) $$L=D+2\lambda \left(X_{a}-\sum_{i=1}^{n^{*}}t_{i}x_{i}^{*}\right)\text{.}$$

After simplifying two Eq. (4) and Eq. (6), the new calibration weight is obtained in Eq. (7)

(7) $$t_{i}^{*}=t_{i}+\frac{t_{i}q_{i}y_{i}^{*}}{\sum_{i=1}^{n}t_{i}q_{i}x_{i}^{*2}}\left[X_{a}-\sum_{i=1}^{n^{*}}t_{i}x_{i}^{*}\right]\text{.}$$

The updated weight $t_{i}^{*}$ is put down in Eq. (3).The proposed estimator $T_{PS,a}$ is obtained by Eq. (8)

(8) $$T_{PS,a}=\sum_{i=1}^{n^{*}}t_{i}y_{i}^{*}+\frac{\sum_{i=1}^{n^{*}}t_{i}q_{i}y_{i}^{*}x_{i}^{*}}{\sum_{i=1}^{n^{*}}t_{i}q_{i}x_{i}^{*2}}\left[X_{a}-\sum_{i=1}^{n^{*}}t_{i}x_{i}^{*}\right]\text{.}$$

Case 1 If we put down $q_{i}=1/x_{i}^{*}$ in Eq. (8). It reduces to the synthetic type ratio estimator in Eq. (9)

(9) $$T_{RS,a}=\frac{\sum_{i=1}^{n^{*}}t_{i}y_{i}^{*}}{\sum_{i=1}^{n^{*}}t_{i}x_{i}^{*}}X_{a}$$

Expectation of the estimator is $E\left(T_{RS,a}\right)=E\left(\frac{\sum_{i=1}^{n^{*}}t_{i}y_{i}^{*}}{\sum_{i=1}^{n^{*}}t_{i}x_{i}^{*}}X_{a}\right)\neq Y_{a}$.

$T_{RS,a}$ is biased estimator. But, for large sample $T_{RS,a}$ is an asymptotically unbiased estimate $Y_{a}$ because a supportive variable weight total of population $\sum_{i=1}^{n^{*}}t_{i}x_{i}^{*}$ near to total of ath domain $X_{a}$ of the supportive variable, it means $\left(\frac{X_{a}}{\sum_{i=1}^{n^{*}}t_{i}x_{i}^{*}}\right)≅\left(\frac{Y_{a}}{\sum_{i=1}^{n^{*}}t_{i}y_{i}^{*}}\right)$.

Hence, $E\left(T_{RS,a}\right)≅Y_{a}$.

Further, we obtained the variance of synthetic type ratio estimator $T_{RS,a}$ for a^th^ domain in Eq. (10)

(10) $$Var\left(T_{RS,a}\right)≅\frac{\sum_{i=1}^{n^{*}}t_{i}^{2}\left(1-f_{i}\right)}{n_{i}^{*}}s_{e_{i}\;}^{2}$$

where, $s_{e_{i}\;}^{2}=\frac{\sum_{i=1}^{n^{*}}e_{i}^{2}}{\left(n^{*}-1\right)}$, also error of i^th^ sample can be written as $e_{i}^{2}=y_{i}^{*}-y_{a}-b\left(x_{i}^{*}-x_{a}\right)$ and $b=\frac{\sum_{i=1}^{n^{*}}t_{i}y_{i}^{*}}{\sum_{i=1}^{n^{*}}t_{i}x_{i}^{*}}$. Asymptotic variance of the proposed estimator $T_{PS,a}$ can be written in the following Eq. (11)

(11) $$Var\left(T_{PS,a}\right)≅\frac{\sum_{i=1}^{n^{*}}D_{i}t_{i}^{*2}}{t_{i}}s_{e_{i}\;}^{2}$$

where, $D_{i}=\frac{t_{i}^{2}\left(1-f_{i}\right)}{n_{i}}$ and $t_{i}^{*}$ is the weight in the proposed estimator.

Case II The generalized form of the proposed estimator would be change [5]. The asymptotic variance of synthetic type of regression estimator for total of domain is given as Eq. (12)

(12) $$Var_{w}\left(T_{PS,a}\right)≅{\left(\frac{X_{a}}{x_{i}^{*}}\right)}^{g}\frac{\sum_{i=1}^{n^{*}}t_{i}^{2}\left(1-f_{i}\right)}{n_{i}^{*}}s_{e_{i}\;}^{2}$$

It can be written into $Var\left(T_{RS,a}\right)$ which is given by Eq. (13)

(13) $$Var\left(T_{PS,a}\right)≅{\left(\frac{X_{a}}{x_{i}^{*}}\right)}^{g}Var\left(T_{RS,a}\right)$$

Here, the explanation of synthetic is given in Eq. (14)

(14) $${\left(\frac{X_{a}}{x_{i}^{*}}\right)}^{g}=1-gX_{a}^{-1}\left(x_{i}^{*}-X_{a}\right)+\frac{g\left(g+1\right)}{2}{\left[X_{a}^{-1}\left(x_{i}^{*}-X_{a}\right)\right]}^{2}+O_{p}\left(n^{-3/2}\right)\text{.}$$

If we write g = 2 in Eq. (13), the estimator reduced to acombine synthetic type ratio estimator for domain total in Eq. (15)

(15) $$Var\left(T_{PS,a}\right)≅Var\left(T_{RS,a}\right)-Var\left(T_{RS,a}\right)\left[2X_{a}^{-1}\left(x_{i}^{*}-X_{a}\right)+3{\left[X_{a}^{-1}\left(x_{i}^{*}-X_{a}\right)\right]}^{2}-O_{p}\left(n^{-3/2}\right)\right]$$

After simplify the relation of the synthetic and generalized synthetic estimate is given in Eq. (16)

(16) $$\text{And}\;Var\left(T_{PS,a}\right)\leq Var\left(T_{RS,a}\right),\text{if}\;\left(\frac{X_{a}}{x_{i}^{*}}\right)\leq 1\text{.}$$

Now we use the constants $C_{x}$ (coefficient of x) and $\beta_{2}\left(x\right)$. We utilized these constants in the proposed estimator because these are like members of ratio estimator. Four usual approach synthetic type ratio estimators and their corresponding synthetic type ratio estimator using calibration approach in the presence of unit non-response are as in Table (1).

**Table 1:** Various synthetic ratio estimators and calibration synthetic type ratio estimators of a^th^ domain.

	Synthetic ratio estimators	Calibration synthetic type ratio estimators
(1)	$\left(T_{SD,a}\right)=y^{*}\;\left(\frac{X_{a}+C_{x}}{x^{*}+C_{x}}\right)$,	$\left(T_{SD,a}^{*}\right)=\frac{\sum_{i=1}^{n^{*}}t_{i}y_{i}^{*}}{\sum_{i=1}^{n^{*}}t_{i}\left(x^{*}+C_{xi}\right)}\sum_{i=1}^{n^{*}}t_{i}\left(X_{a}+C_{xi}\right)$
(2)	$\left(T_{SK,a}\right)=y^{*}\;\left(\frac{X_{a}+\beta_{2}\left(x\right)}{x^{*}+\beta_{2}\left(x\right)}\right)$,	$\left(T_{SK,a}^{*}\right)=\frac{\sum_{i=1}^{n^{*}}t_{i}y_{i}^{*}}{\sum_{i=1}^{n^{*}}t_{i}\left(x^{*}+\beta_{2}\left(x\right)\right)}\sum_{i=1}^{n^{*}}t_{i}\left(X_{a}+\beta_{2}\left(x\right)\right)$
(3)	$\left(T_{US1,a}\right)=y^{*}\;\left(\frac{X_{a}\beta_{2}\left(x\right)+C_{x}}{x^{*}\beta_{2}\left(x\right)+C_{x}}\right)$,	$\left(T_{US1,a}^{*}\right)=\frac{\sum_{i=1}^{n^{*}}t_{i}y_{i}^{*}}{\sum_{i=1}^{n^{*}}t_{i}\left(\beta_{2}\left(x\right)x^{*}+C_{xi}\right)}\sum_{i=1}^{n^{*}}t_{i}\left(\beta_{2}\left(x\right)X_{a}+C_{xi}\right)$
(4)	$\left(T_{US2,a}\right)=y^{*}\;\left(\frac{C_{x}X_{a}+\beta_{2}\left(x\right)}{x^{*}C_{x}+\beta_{2}\left(x\right)}\right)$,	$\left(T_{US2,a}^{*}\right)=\frac{\sum_{i=1}^{n^{*}}t_{i}y_{i}^{*}}{\sum_{i=1}^{n^{*}}t_{i}\left(C_{x}x^{*}+\beta_{2}\left(x\right)\right)}\sum_{i=1}^{n^{*}}t_{i}\left(C_{x}X_{a}+\beta_{2}\left(x\right)\right)$

For different choice of the positive constants $C_{x}$ and $\beta_{2}\left(x\right)$ we used two Eq. (12) and Eq. (13), we get different synthetic type ratio estimators in Table (2).Where, the first terms of variance are less than one mention in Equation (6). Hence, we can say that the approximate variance synthetic form of ratio estimators of total of the small domain using calibration estimator is smaller to combine synthetic type of ratio estimator for domain total using an auxiliary variable for all the domains. One limitation is that, it is very low data set are available for meeting the indirect method.

**Table 2:** Asymptotic variance of various synthetic ratio estimators with their positive constants of a^th^ domain.

	Positive Constant $\left(q_{i}\right)$	Variance
(1)	$\left(\frac{1}{x^{*}+C_{x}}\right)$,	$\left(T_{SD,a}^{*}\right)≅{\left(\frac{X_{a}+C_{x}}{x^{*}}\right)}^{2}\sum_{i=1}^{n^{*}}\frac{t_{i}^{2}\left(1-f_{i}\right)}{n_{i}^{*}}s_{e\;i}^{2}$
(2)	$\left(\frac{1}{x^{*}+\beta_{2}\left(x\right)}\right)$,	$\left(T_{SK,a}^{*}\right)≅{\left(\frac{X_{a}+\beta_{2}\left(x\right)}{x^{*}}\right)}^{2}\sum_{i=1}^{n^{*}}\frac{t_{i}^{2}\left(1-f_{i}\right)}{n_{i}^{*}}s_{e\;i}^{2}$
(3)	$\left(\frac{1}{x^{*}\beta_{2}\left(x\right)+C_{x}}\right)$,	$\left(T_{US1,a}^{*}\right)≅{\left(\frac{\beta_{2}\left(x\right)X_{a}+C_{x}}{x^{*}}\right)}^{2}\sum_{i=1}^{n^{*}}\frac{t_{i}^{2}\left(1-f_{i}\right)}{n_{i}^{*}}s_{e\;i}^{2}$
(4)	$\left(\frac{1}{x^{*}C_{x}+\beta_{2}\left(x\right)}\right)$,	$\left(T_{US2,a}^{*}\right)≅{\left(\frac{X_{a}C_{x}+\beta_{2}\left(x\right)}{x^{*}}\right)}^{2}\sum_{i=1}^{n^{*}}\frac{t_{i}^{2}\left(1-f_{i}\right)}{n_{i}^{*}}s_{e\;i}^{2}$

## 4 Conclusion

The eminent point of the work is, how to use both constants $C_{x}$ and $\beta_{2}\left(x\right)$ in the various synthetic estimators for estimating domain total? In Case 1 synthetic ratio estimator whenever, Case 2 explain generalized form of the Case 1. The four discussed estimators are superior over Case 1 estimators under condition of $\left(\frac{X_{a}}{x^{*}}\right)<1$. The performance of the investigated estimator is to be better than ratio synthetic estimates when, the information of population meets to the information of the domain and both constants coefficient of variation and kurtosis. The domain total of intended variable is efficient if supportive variable domain total and supportive variable of population. If we developed some other estimators with use of $C_{x}$ and $\beta_{2}\left(x\right)$, it will converges to proposed estimators. We theoretically found that the proposed estimators of domain total have superior over traditional synthetic ratio estimators of domain total. It has also seen that the calibration approach is a better option over traditional approach.

## 5 Application

The bestow work may be used in small group of persons where low or least available of natural resources. The quality of socio-economic conditions degraded in the diverse regions.

## Data availability statement

Data will be made available on request.

## Future plan

The bestow work can extend in two stage sampling, two auxiliary and stratified random sampling for small domain.

## CRediT authorship contribution statement

**Abdullah Mohammed Alomair:** Writing – review & editing, Writing – original draft, Visualization, Validation, Software, Resources, Project administration, Methodology, Funding acquisition. **Ashutosh Ashutosh:** Writing – review & editing, Writing – original draft, Methodology.

## Declaration of competing interest

The authors declare that they have no known competing financial interests or personnel relationships that could have appeared to influence the work reported in this paper.
